# Brain MRI findings in severe COVID-19 patients: a meta-analysis

**DOI:** 10.3389/fneur.2023.1258352

**Published:** 2023-10-12

**Authors:** Montek S. Boparai, Benjamin Musheyev, Wei Hou, Mark F. Mehler, Tim Q. Duong

**Affiliations:** ^1^Renaissance School of Medicine at Stony Brook University, Stony Brook, NY, United States; ^2^Department of Radiology, Montefiore Medical Center and Albert Einstein College of Medicine, Bronx, NY, United States; ^3^Department of Neurology, Montefiore Health System and Albert Einstein College of Medicine, Bronx, NY, United States

**Keywords:** COVID-19, magnetic resonance imaging, brain, neurocognitive, cerebral microbleeds (CMB), infarct

## Abstract

**Introduction:**

Neurocognitive symptoms and dysfunction of various severities have become increasingly recognized as potential consequences of SARS-CoV-2 infection. Although there are numerous observational and subjective survey-reporting studies of neurological symptoms, by contrast, those studies describing imaging abnormalities are fewer in number.

**Methods:**

This study conducted a metanalysis of 32 studies to determine the incidence of the common neurological abnormalities using magnetic resonance imaging (MRI) in patients with COVID-19.

**Results:**

We also present the common clinical findings associated with MRI abnormalities. We report the incidence of any MRI abnormality to be 55% in COVID-19 patients with perfusion abnormalities (53%) and SWI abnormalities (44%) being the most commonly reported injuries. Cognitive impairment, ICU admission and/or mechanical ventilation status, older age, and hospitalization or longer length of hospital stay were the most common clinical findings associated with brain injury in COVID-19 patients.

**Discussion:**

Overall, the presentation of brain injury in this study was diverse with no substantial pattern of injury emerging, yet most injuries appear to be of vascular origin. Moreover, analysis of the association between MRI abnormalities and clinical findings suggests that there are likely many mechanisms, both direct and indirect, by which brain injury occurs in COVID-19 patients.

## Introduction

Coronavirus Disease 2019 (COVID-19) (1, 2) characteristically involves multiple organ systems, including the central and peripheral nervous system. SARS-CoV-2 infection has been associated with a range of neurological phenomena, which are still incompletely understood. Severe acute neurological events include ischemic stroke, intracranial hemorrhage, encephalopathy, seizure disorders, extrapyramidal syndromes, neuromuscular pathologies, various immune-mediated neuroinflammatory disorders, and dysautonomias (3). In this context, neurocognitive symptoms and dysfunction of various severities have become increasingly recognized as potential consequences of SARS-CoV-2 infection. While brain dysfunction might be attributed to the effects of critical care illness among hospitalized patients, emerging data indicate that brain effects are also prevalent among less severely ill, non-hospitalized and even mildly symptomatic patients (4). Although there are numerous observational and subjective survey-reporting studies of neurological symptoms, by contrast, those studies describing imaging abnormalities are fewer in number.

This study conducted a metanalysis to determine the incidence of the common neurological abnormalities using magnetic resonance imaging (MRI) in patients with COVID-19. This study expands on a previous metanalysis of COVID-19 neuroimaging performed early in the pandemic (5) providing a more contemporary and elaborate analysis. We also present the common clinical findings associated with MRI abnormalities.

## Methods

### Eligibility criteria and evidence search

**Figure 1:**
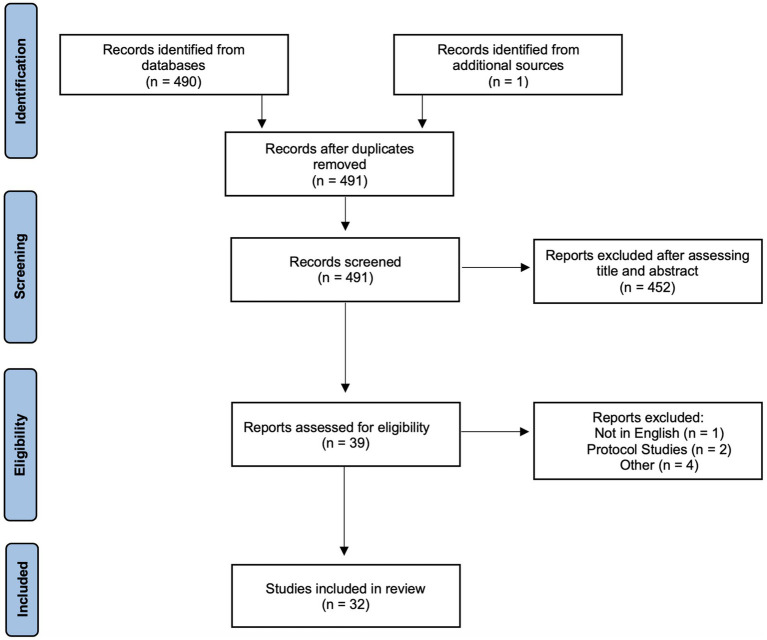
PRISMA diagram illustrating the study eligibility criteria.

Using Preferred Reporting Items for Systematic Reviews and Meta-analyses (PRISMA), we conducted a systematic review of studies which reported neurological MRI findings in COVID-19 patients (Figure 1). A PubMed, Embase and Google Scholar database search from January 1, 2020, to June 17, 2022, was performed. Additional papers found outside of these searches were added at the authors’ discretion. The search parameters can be found in the Supplementary Information. Cross-sectional, case–control, and cohort studies were included in the analyses. Studies that were excluded included: (1) case reports, case series, review papers, and conference abstracts; (2) papers not written in English; (3) protocol papers, letters to the editor, preprint papers, and healthcare provider surveys without data; and (4) papers that did not use MRI as a data metric.

The title and abstract of papers after the initial search were assessed by two independent reviewers, MB and BM, and only studies approved by both reviewers were included. Disputes regarding the inclusion of a paper were decided by a third reviewer, TD.

### Data collection and analysis

Study characteristics, including author, study type, origin, sample size and other qualitative findings were manually collected. The incidence of any brain MRI abnormality as well as the incidence of common specific and subspecific brain MRI abnormalities after SARS-CoV-2 infection were collected manually.

## Results

The initial search resulted in 491 articles with no duplicates. After assessing the title and abstract, 452 papers were removed. An additional seven papers which did not meet the inclusion criteria were removed after assessing the entire paper. Thirty-two papers were included in the final study. Table 1 summarizes the study characteristics and main findings.

**Table 1:** Study characteristics.

Paper	Author	Study design	Country	Date	Pts (N)	M:F	Follow-up time	Main findings
Neurological complications in critical patients with COVID-19	Abenza-Abildúa (6)	Retrospective	Spain	July 29 2020	30	72:28	Acute (time not given)	COVID-19 was definite cause neurological symptoms in 20% of patients. Symptoms were not associated with imaging findings.
Cerebral Microbleeds and Leukoencephalopathy in Critically Ill Patients With COVID-19	Shashank Agarwal (7)	Retrospective	USA	July 8 2020	115	N/A	LE or CMB = 27 (10.3) days No LE or CMB = 10.6 (12.9) days	30.4% of patients had CMB and LE on neuroimaging. These findings were associated with lower neurological status (GCS).
Retrospective Observational Study of Brain MRI Findings in Patients with Acute SARS-CoV-2 Infection and Neurologic Manifestations	Lydia Chougar (8)	Cross-Sectional	France	July 7 2020	73	66:34	22.3 ± 15.7 days	59% of patients had an abnormal MRI finding. The pattern of WM enhancement and basal ganglia involvement seen in COVID-19 is unlike any other previously characterized condition/pathology.
Unusual Microbleeds in Brain MRI of Covid-19 Patients	Aikaterini Fitsiori (9)	Retrospective*	Switzerland	June 24 2020	9	78:22	Acute (time not given)	MRI revealed an atypical predilection for the corpus callosum. Severe hypoxemia and ventilation status was common among all patients with MRI abnormalities.
Delirium and encephalopathy in severe COVID-19: a cohort analysis of ICU patients	Julie Helms (10)	Prospective	France	July 26 2020	28	N/A	Acute (time not given)	Brain lesions and perfusions abnormalities seen on MRI strengthen the case for a COVID-19 associated encephalopathy and/or encephalitis.
Brain MRI Findings in Patients in the Intensive Care Unit with COVID-19 Infection	Sedat G. Kandemirli (11)	Retrospective	Turkey	May 5 2020	27	78:22	Acute (time not given)	44% of patients who underwent brain MRI had acute findings. The main differential diagnoses for the pattern of injury seen are encephalitis and hypoxia.
Nervous System Involvement in Coronavirus Disease 2019:	Stefanos Klironomos (12)	Retrospective	Sweden	July 30 2020	43	N/A	Median 34 days	Intra axial abnormalities, leukoencephalopathy were common. Pattern of imaging is similar to endotheliopathy and microthrombosis.
Neurologic and neuroimaging findings in patients with COVID-19	Stephane Kremer (13)	Retrospective	France	June 9 2020	64	67:33	Acute (can calculate if needed)	Imaging abnormalities were heterogenous in nature, and associated clinical symptoms were also heterogenous. Three clinic radiological profiles were identified: ischemic stroke, LME, and encephalitis.
Brain MRI Findings in Severe COVID-19: A Retrospective Observational Study	Stephane Kremer (14)	Retrospective	France	June 16 2020	190	81:19	Acute (time not given)	54% of patients experienced COVID-19 related hemorrhagic lesions (macro and micro). These were associated with worse neurological status. 43% showed signal abnormalities in the medical temporal lobe.
Increase in Ventricle Size and the Evolution	Shashank Agarwal (15)	Retrospective	USA	February 6 2021	21	86:14	First MRI = 22 [14–30] Second MRI = 49 [39–60]	Increased ventricle size between the two MRIs. Some patients showed worsening of WM changes on second MRI, some showed improved, but the majority remained stable.
Brain MRI in SARS-CoV-2 pneumonia patients with newly developed neurological manifestations suggestive of brain involvement	Batil Alonazi (16)	Retrospective	Saudi Arabia	October 5 2021	46	28:72	5 days	MRI abnormalities were more common in patients who presented with non-focal neurological manifestation or had a lower GCS.
Clinical and Radiological Profiles of COVID-19 Patients with Neurological Symptomatology: A Comparative Study	Maria de Fatima Viana Vasco Aragao (17)	Retrospective	Brazil	April 27 2021	35	57:43	Acute (time not given)	Neuroimaging evaluation of olfactory bulbs showed lesions in 12/12 patients. Given this, anosmia may be considered a central neurological symptom rather than a flu-like symptom.
Collicular Hyperactivation in Patients with COVID-19: A New Finding on Brain MRI and PET/CT	Chammas (18)	Retrospective	France	March 11 2021	72	N/A	Acute (30 days) Follow-up at 3-month	17% of patient had hyperperfusion of the lower colliculi on acute imaging which was less pronounced at follow-up.
Susceptibility-weighted imaging reveals cerebral microvascular injury in severe COVID-19	John Conklin (19)	Retrospective	USA	January 4 2021	16	N/A	Acute (time not given)	Hemorrhagic and ischemic microvascular lesions are common in COVID-19 patients with neurological deficits. These imaging findings were confirmed in one patient at autopsy.
Coronavirus Disease (COVID-19)-Related Disseminated Leukoencephalopathy: A Retrospective Study of Findings on Brain MRI	Colbey W. Freeman (20)	Retrospective	USA	August 31 2020	59	N/A	Acute (time not given)	10.2% of patients had findings consistent with the authors definition of COVID-19-related disseminated leukoencephalopathy.
Yield of Head Imaging in Ambulatory and Hospitalized Patients With SARS-CoV-2: A Multi-Center Study of 8,675 Patients	Melanie R. F. Greenway (21)	Retrospective	USA	December 16 2020	23	58:42	Acute (0–30 days)	Rate of brain imaging and cerebrovascular events was low. No association between rate of cerebrovascular events and disease severity was found.
Brain MRI and neuropsychological findings at long-term follow-up after COVID-19 hospitalization: an observational cohort study	Lovisa Hellgren (22)	Ambidirectional	Sweden	October 12 2021	35	80:20	7 months post-admission	25/35 patients had an abnormal MRI at the 7-month follow up. Increased age and a higher premorbid function category were associated with an abnormal brain MRI at follow up.
Association of Clinical, Biological, and Brain Magnetic Resonance Imaging Findings With Electroencephalographic Findings for Patients With COVID-19	Virginie Lambrecq (23)	Retrospective	France	March 15 2021	57	N/A	Acute (time not given)	72% of patient presented with an abnormal brain MRI. Patient with COVID-19 encephalopathy were more likely to present with WM-enhancing lesions on MRI.
Abnormal MRI findings of the orbital or visual pathways in patients with severe COVID-19: Observations from the French multicenter COVID-19 cohort	Augustin Lecler (24)	Retrospective	France	October 18 2021	129	67:33	Acute (time not given)	13% of patients with severe COVID-19 had abnormal findings of the orbit or visual pathway on brain MRI. Visual impairments may go unnoticed in patients under sedation due to COVID-19.
Cerebral vasculitis of medium-sized vessels as a possible mechanism of brain damage in COVID-19 patients	Francois Lersy (25)	Retrospective	France	May 3 2021	69	67:33	Acute (can calculate time)	16% of COVID-19 patients had a brain MRI consistent with cerebral vasculitis. Cerebral vasculitis was significantly less common in patients without SARS-CoV-2 infection.
Critical illness-associated cerebral microbleeds for patients with severe COVID-19: etiologic hypotheses	Francois Lersy (26)	Retrospective	France	November 8 2020	80	84:16	26 (20–31) days with WM microhemorrhages. 12 (6–18) days without WM microhemorrhages	24% of patients presented with COVID-19 associated cerebral microbleeds (CIAM). Patients with CIAM presented with worse neurological status than those without CIAM.
Central Nervous System Injury in Patients With	Edith Fabiola Mendez Elizondo (27)	Retrospective	Mexico	September 17 2021	47	N/A	Acute (time not given)	13% of patients with COVID-19 were found to have microbleeds. Presentation of patients was heterogenous with various brain pathologies seen on MRI.
Distinct pattern of microsusceptibility changes on brain magnetic resonance imaging (MRI) in critically ill patients	Majda M. Thurnher (28)	Retrospective	Austria	March 2 2021	48	50:50	Acute (time not given)	A distinct SWI susceptibility (microbleed) pattern is seen in patients who undergo ECMO. Pattern on injury was diffuse without relation to any specific vascular territory.
Long COVID-19: Objectifying most self-reported neurological symptoms	Julia Bungenberg (29)	Cross-sectional	Germany	December 15 2021	42	N/A	29.3 weeks (3.3–57.9)	MRI findings were within normal clinical references despite deficiencies in cognitive performance. This may indicate that even MRI is not sensitive enough to detect subtle brain changes in COVID-19.
Cognitive, EEG, and MRI features of COVID-19 survivors: a 10-month study	Giordano Cecchetti (30)	Retrospective	Italy	February 22 2022	36	69:31	2 months	Patients with COVID-19 had greater WM hyperintensities in the right frontal and eight parietooccipital lobe compared to healthy controls. This finding corelated with worse memory function.
Evolution of Neuroimaging Findings in Severe COVID-19 Patients with Initial Neurological Impairment: An Observational Study	François Lersy (31)	Retrospective	France	April 26 2022	31	74:26	Acute 3 months 6 months	Brain MRI abnormalities typically regress (normalize) or remain stable over time. New complications months after COVID-19 are rare and their relation to COVID-19 is difficult to discern.
Cerebral Microbleeds Assessment and Quantification in COVID-19 Patients With Neurological Manifestations	Angela Napolitano (32)	Retrospective	Italy	April 7 2022	63	62:38	61 days	22% of patients had evidence on CMBs on MRI. The pattern of CMB was callosal and juxtacortical which has been previously seen in patients requiring mechanical ventilation;
Early postmortem brain MRI findings in COVID-19 non-survivors	Tim Coolen (33)	Prospective	Belgium	October 6 2020	19	74:26	13.67 (2.07–23.75) hours postmortem	Hemorrhagic, olfactory, and PRES-related brain lesion were common findings in deceased COVID-19 patients. No brainstem abnormalities were observed, arguing against brainstem contribution to respiratory distress.
Disorders of Consciousness Associated With COVID-19	David Fischer (34)	Prospective	USA	January 18 2022	12	42:58	Acute (exact time not given)	Microhemorrhages and leukoencephalopathy 55 and 45% of patients, respectively. Patients with severe COVID-19 are likely to have less brain interconnectivity than healthy controls.
Neurologic manifestations associated with COVID-19: a multicentre registry	Elodie Meppiel (35)	Retrospective	France	Nov 13 2020	222	61:39	24 days	Infarcts, encephalitis, and encephalopathy were the most common imaging abnormalities reported. Overall, neurological manifestations of COVID-19 are vast and heterogenous.
Neuroimaging Findings of Hospitalized Covid-19 Patients: A Canadian Retrospective Observational Study	Vibeeshan Jegatheeswaran (36)	Retrospective	Canada	April 21 2021	422	54:46	94 days	The main MRI findings were macrohemorrhages, SWI abnormalities, and acute ischemia. ICU patients were more likely to have positive imaging findings.
Brain Imaging of Patients with COVID-19: Findings at an Academic Institution during the Height of the Outbreak in New York City	Lin (37)	Retrospective	USA	July 17 2020	278	59:41	Acute (time not given)	Infarcts (acute and subacute) were the most common findings on brain MRI. 12% of patients had cranial nerve abnormalities and 6% had critical illness-associated microbleeds.

### Incidence of brain MRI abnormalities

**Figure 2:**
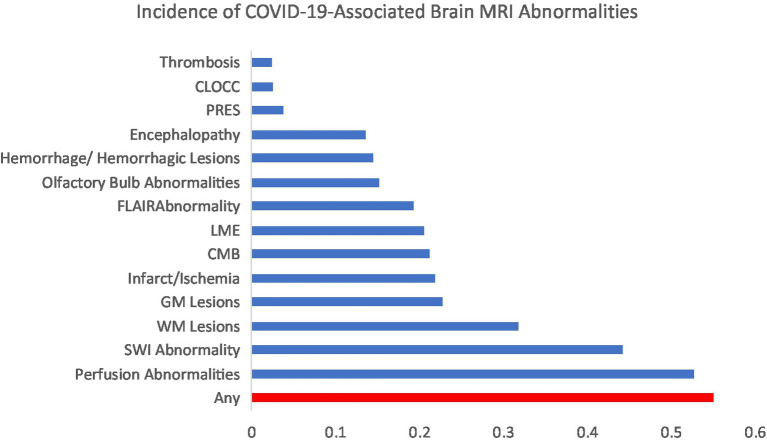
Incidence of the most common COVID-19-associated brain MRI abnormalities. CLOCC, cytotoxic lesions of the corpus callosum; PRES, posterior reversible encephalopathy syndrome; FLAIR, fluid-attenuated inversion recovery; LME, leptomeningeal enhancement; CMB, cerebral microbleeds; GM, gray matter; WM, white matter; SWI, susceptibility weighted imaging.

Figure 2 illustrates the incidence of brain MRI abnormalities after SARS-CoV-2 infection. The incidence of any brain MRI abnormality after SARS-CoV-2 infection was 55% (461/837 patients). The most common brain abnormalities in order of incidence were perfusion abnormalities (53%), susceptibility weighted imaging (SWI) abnormality (44%), white matter lesions (32%), gray matter lesions (23%), infarct/ischemia (22%), cerebral microbleeds (CMB; 21%), leptomeningeal enhancement (LME; 21%), fluid attenuated inversion recovery (FLAIR) abnormality (19%), olfactory bulb abnormalities (15%), hemorrhage (15%), encephalopathy (14%), posterior reversible encephalopathy syndrome (PRES; 4%), cytotoxic lesions of the corpus callosum (CLOCC; 3%) and thrombosis (2%). Subspecific information regarding brain MRI abnormalities are presented in Table 2.

**Table 2:** Common neurological MRI abnormalities in COVID-19 patients.

MRI abnormality		No. of Pts (%)
Infarct		152/706 (22)
	Acute	65/378 (17)
	Subacute	5/82 (6)
	Chronic	21/188 (11)
	Lacunar	14/55 (26)
	Territorial	20/146 (14)
	Watershed	9/75 (12)
	Subcortical	1/9 (11)
CMB		195/920 (21)
	Diffuse	37/209 (18)
	Lobar	35/171 (21)
	Deep WM	25/222 (11)
	Subcortical WM	33/171 (19)
	Corpus callosum	48/302 (16)
	Pons/cerebellum	15/212 (7)
	Basal ganglia	4/83 (5)
Perfusion abnormalities		59/112 (53)
	Seizure Related	9/46 (12)
	2^°^ to Ischemic Lesions	4/46 (5)
	Hypoperfusion	19/40 (48)
	Hyperperfusion	4/40 (10)
WM Lesions		87/274 (32)
	Periventricular	46/104 (44)
	Juxtacortical	27/62 (44)
	Subcortical	17/21 (81)
	Corpus callosum	11/41 (27)
	Middle cerebellar peduncles	7/41 (17)
	Cerebellum	4/21 (19)
	Brainstem	6/21 (29)
	Basal ganglia	1/42 (2)
	Precentral gyrus	6/21 (29)
FLAIR abnormality		53/274 (19)
	Confluent	12/262 (5)
	Non-confluent	23/262 (9)
	Frontal lobe	4/27 (15)
	Parietal lobe	3/27 (11)
	Occipital lobe	5/50 (10)
	Temporal lobe	2/50 (4)
	Medial temporal lobe	20/262 (8)
	Corpus callosum	7/249 (3)
	Middle cerebellar peduncle	4/268 (2)
	Brainstem	4/59 (7)
SWI abnormality		73/165 (44)
	Cortical	9/16 (56)
	Subcortical	18/73 (25)
	Juxtacortical	17/56 (30)
	Deep and Periventricular WM	10/33 (30)
	Cerebellum	6/16 (38)
	Thalami	5/16 (31)
	Basal ganglia	1/64 (2)
	Brainstem	3/16 (19)
	Corpus callosum	34/120 (28)
	Pons	1/48 (2)
		
Thrombosis		4/164 (2)
	Venous	3/77 (4)
	Arterial	1/47 (2)
Hemorrhage		63/436 (15)
GM Lesions		13/57 (23)
Leptomeningeal enhancement		50/244 (21)
Encephalopathy		98/722 (14)
CLOCC		6/235 (3)
PRES		9/237 (4)
Olfactory bulb abnormalities		24/158 (15)

CMB, cerebral microbleeds; WM, white matter; FLAIR, fluid-attenuated inversion recovery; SWI, susceptibility weighted imaging; GM, gray matter; CLOCC, cytotoxic lesions of the corpus callosum.

The incidence of acute infarcts (17%) was more common than chronic (11%) and subacute infarcts (6%). Lacunar infarcts were the most common (26%) followed by territorial arterial infarcts (14%) and watershed infarcts (12%). Cortical stroke was not reported in any studies whereas the incidence of subcortical stroke was found to be 11% in one study.

The incidence of lobar CMBs (21%) was slightly higher than diffuse CMBs (18%). The incidence of CMBs in the subcortical and deep WM was 19 and 11%, respectively. The most commonly affected subcortical structures were the corpus callosum (16%), pons/cerebellum (7%), and basal ganglia (5%).

Hypoperfusion abnormalities (48%) were more common than hyperperfusion abnormalities (10%). The incidence of seizure-related perfusion abnormalities was 12%. Perfusion abnormalities secondary to ischemic lesions was 5%.

The incidence of subcortical WM changes was 81%. The incidence of periventricular and juxtacortical WM changes was 44% each. The most common sites for WM changes were the brainstem (29%), precentral gyrus (29%), corpus callosum (27%), cerebellum (19%), middle cerebellar peduncles (17%), and basal ganglia (2%).

Non-confluent FLAIR abnormalities (9%) were more common than confluent ones (5%). The most common locations were the frontal lobe (15%), parietal lobe (11%), occipital lobe (10%), medial temporal lobe (8%), brainstem (7%), temporal lobe (4%), corpus callosum (3%), and middle cerebellar peduncles (2%).

The incidence of cortical, juxtacortical, and subcortical on DWI was 56, 30, and 25%, respectively. A combined incidence of deep and periventricular WM SWI abnormality (30%) was reported in one study. The most common sites for SWI abnormalities were cerebellum (38%), thalami (31%), corpus callosum (28%), brainstem (19%), basal ganglia (2%), and pons (2%).

The incidence of venous thrombosis (4%) was marginally higher than arterial thrombosis (2%).

**Figure 3:**
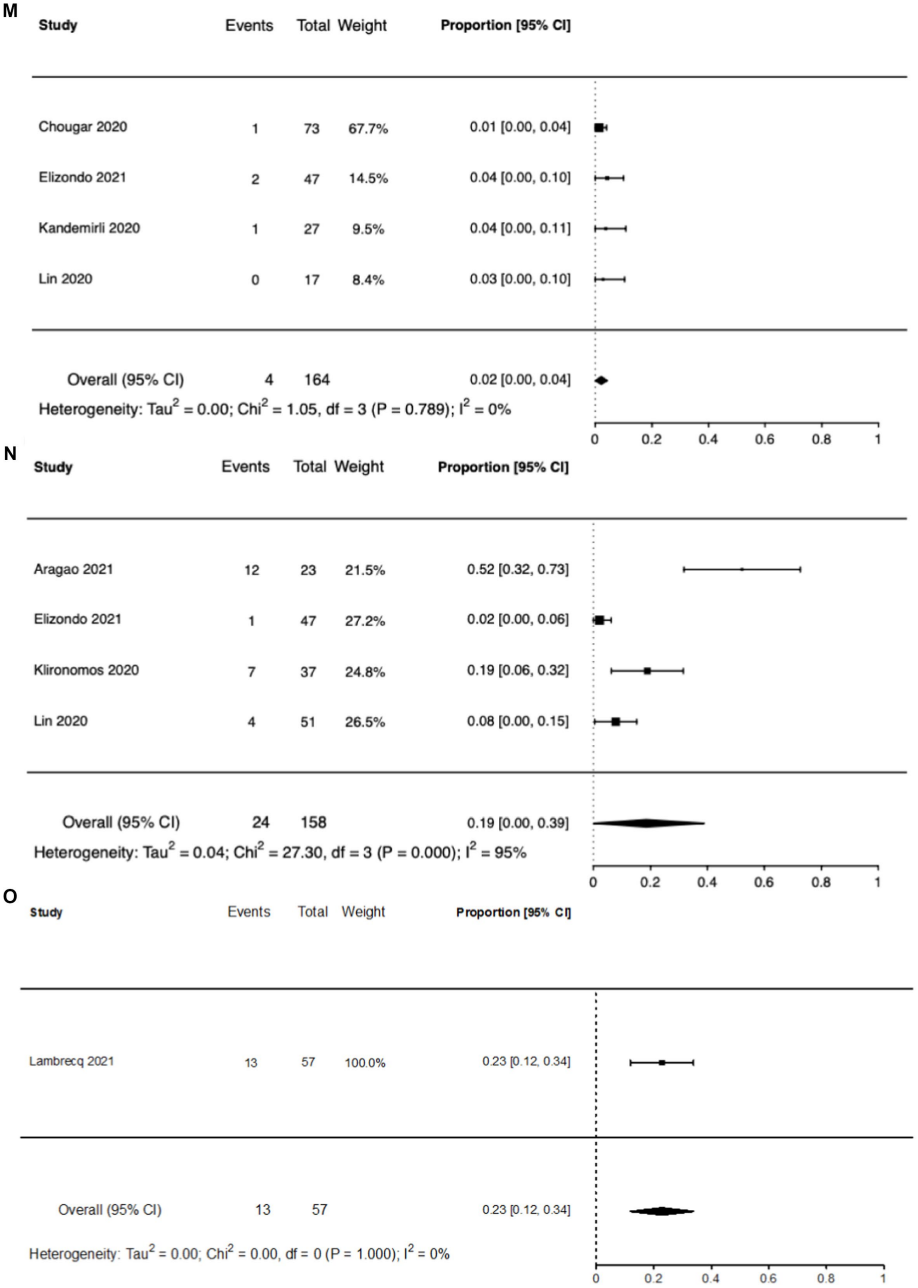
Forest plots of reported brain MRI abnormalities. **(A)** Any brain MRI abnormality. **(B)** Cerebral microbleeds. **(C)** Encephalopathy. **(D)** Hemorrhage. **(E)** Infarct. **(F)** White matter lesions. **(G)** FLAIR abnormality. **(H)** SWI Abnormality. **(I)** Posterior reversible encephalopathy syndrome. **(J)** Leptomeningeal enhancement. **(K)** Cytotoxic lesion of the corpus callosum. **(L)** Perfusion abnormalities. **(M)** Thrombosis. **(N)** Olfactory bulb abnormalities. **(O)** Gray matter lesions.

Figure 3 shows the inter-study heterogeneity for the commonly reported brain MRI abnormalities.

### Clinical measures associated with MRI abnormalities

Twelve studies in this review reported a statistical (*p* < 0.05) association between at least one clinical datapoint and an MRI abnormality. The most commonly reported associations were cognitive impairment (6), followed by ICU and/or mechanical ventilation status (5), older age (4 studies), hospitalization or longer length of hospital stay (4), and ARDS (2). The most commonly reported laboratory marker was elevated WBC count (3), higher D-Dimer (2), higher creatinine (2) and decreased hemoglobin (2). Table 3 qualitatively outlines the MRI abnormality and the associated clinical parameter for each of the 12 studies.

**Table 3:** Clinical findings associated with MRI abnormalities.

Author	MRI abnormality	Association(s)	Non-association(s)
Agarwal (2020) (7)	CMB and/or encephalopathy	Age (higher), GCS at time of MRI (lower), Ventilation duration (higher), Moderate, severe hypoxemia, Length of hospital stay (higher), Time from admission to MRI (higher), mRS at discharge (higher), Peak INR (higher), Peak D-dimer (higher), Platelet count nadir (lower)	Sex, BMI, Hyperlipidemia, Diabetes Mellitus, Hypertension, Admission platelets Admission D-dimer, Admission Fibrinogen Admission INR
Chougar (2020) (8)	≥5 microhemorrhages, Microhemorrhage with corpus callosum involvement, Perfusion abnormalities, Multifocal WM lesions, Basal ganglia lesion	ICU admission	–
Kremer (2020) (14)	Hemorrhagic lesions	ARDS, ICU admission, Time from symptom onset to brain MRI (higher), Abnormal wakefulness in ICU, WBC count (higher), Hemoglobin (lower), Blood urea (higher)	Sex, Age, Oxygen therapy, Death, Neurological manifestations except abnormal wakefulness, Lymphocyte count, Platelet count, CRP, Ferritin, ALT, AST, Creatinine, PTT, Fibrinogen, D-dimer, CSF analysis
Kremer (2020) (13)	Ischemic stroke	Age (older), Corticospinal tract involvement	Sex, Headache, Seizure, Anosmia, Ageusia, Disorder of consciousness, Confusion, Oxygen therapy, Death
	Encephalitis	Age (younger), ARDS	
	LME	Agitation	
Chammas (2021) (18)	Hyperperfusion of the colliculi	Admission WBC count (higher), Seizures, LME	Severity of disease
Hellgren (2021) (22)	Any abnormal brain MRI	Age (higher), Premorbid function category (higher), Visuospatial Index (lower)	Sex, Days in hospital, ICU care, Mechanical ventilation, CRP, D-dimer, Neurocognition, Fatigue, Depression, Anxiety
Lersy (2020) (26)	WM microhemorrhages	ICU duration (higher), Hospital duration (higher), Time between intubation and MRI (higher), Disturbance of consciousness, Confusion, Agitation, Urea (higher), D-Dimer (higher), Creatinine (higher), Dialysis	Sex, Age, Cardiovascular Risk Factors, Seizures, Corticospinal tract involvement, Pathological wakefulness when sedatives were stopped
Lersy (2020) (25)	Imaging consistent with cerebral vasculitis	Age (higher)	Sex, Diabetes, Hypertension, Hyperlipidemia, Smoking, Obesity
Bungenberg (2021) (29)	CMB	Hospitalization, Worse visuospatial processing	Age
Cecchetti (2022) (30)	Higher WM volume in left frontal region	Cardiovascular risk factors	–
	Higher WM volume in left parieto-occipital region	Poor memory and recall performance	–
Napolitano (2022) (32)	CMB	Hospitalization, Time to MRI (higher), Invasive mechanical ventilation, Leukoencephalopathy, Inflammatory CSF, WBC (higher), Lymphocytes (higher), Hemoglobin (lower), CRP (lower), Procalcitonin (lower), PT (lower), Fibrinogen (lower)	Sex, Age, Dyslipidemia, Heart disease, Diabetes, Hypertension, COPD, Confusion, Visual Impairment, Stroke, Seizure, Anosmia, Neuropathy, Platelet count, LDH, aPTT, D-dimer

*MRI and/or CT.

## Discussion

In this metanalysis, we report the pooled incidence of the commonly reported brain MRI abnormalities in patients with COVID-19. The pooled incidence of any brain MRI abnormality was found to be 55% [Proportion = 0.65; 95% CI = 54–76%; *I*^2^ = 94%]. The five most commonly studied abnormalities were WM lesions [Proportion = 0.39; 95% CI = 11–66%; *I*^2^ = 99%], cerebral microbleeds [Proportion = 0.29; 95% CI = 16–38%; *I*^2^ = 95%], hemorrhage [Proportion = 0.16; 95% CI = 9–22%; *I*^2^ = 74%], infarct [Proportion = 0.18; 95% CI = 11–21%; *I*^2^ = 65%], and encephalopathy [Proportion = 0.12; 95% CI = 3–18%; *I*^2^ = 94%]. Perfusion abnormalities (53%) and SWI abnormalities (47%) were the two brain abnormalities with the highest incidence. The most reported clinical characteristic and laboratory value with a statistically significant association with at least one brain MRI abnormality was cognitive impairment and elevated WBC count, respectively. Together, these results show that brain MRI abnormalities after SARS-CoV-2 infection are common and that clinical associations may provide insight into identifying at-risk patients as well as possible combinatorial and intersectional mechanisms of brain injury in COVID-19.

There is considerable heterogeneity in the results reported in this meta-analysis, a finding which is similar to a smaller meta-analysis performed earlier in the pandemic (5). There are a few reasons for the observed heterogeneity. First, there is substantial interstudy variation in patient populations, study designs, and end-outcomes. Second, the neurological presentation of COVID-19 is itself very heterogenous. Unlike other pathogens, such as Lyme Disease and Herpes Simplex Virus (38), which may reveal distinct patterns of injury on brain MRI, there is no unanimous pattern of brain injury with SARS-CoV-2, likely due to multifactorial and synergistic mechanisms of direct and indirect injury responses. Indirect effects include respiratory distress, sepsis, hypoxia, cardiovascular distress, host-mediated proinflammatory responses, hypercoagulation, amongst many others. Whether the heterogeneity seen in our study is the result of interstudy variation or the result of the inherent diversity of SARS-CoV-2-mediated brain injury remains to be seen.

The incidence of any brain MRI abnormality was found to be 55%. This number is likely greatly inflated given that the majority of studies included in this meta-analysis looked at patients that had severe COVID-19. Indeed, a previous study found the rate of brain MRI abnormalities to be less than 1% (51/5430) when looking at all COVID-19 patients regardless of severity (39), although this is likely an underestimation given that not all patients in the aforementioned study were referred for MRI analysis. Regardless, the findings of our meta-analysis are likely more useful to the clinician managing a critically ill COVID-19 patient in the ICU than the clinician managing a milder form of the disease.

Clinicians should be aware that the presentation of brain injury in COVID-19 can be diverse, although the majority of brain abnormalities in COVID-19 appear to be cerebrovascular events. The two injuries with the highest prevalence are perfusion abnormalities and SWI abnormalities, the latter of which usually indicates a cerebral microbleed and/or calcification (40). Infarcts, hemorrhages, cerebral microbleeds, and thrombosis are also of cerebrovascular origin. On the other hand, olfactory bulb lesions are likely exclusively associated with nerve damage (17). The source of the rest of the abnormalities can vary.

The question of how much COVID-19 contributes to abnormal brain MRI findings is unclear, especially since the patients indicated for brain MRI are often the sickest patients with several comorbidities that may present as confounders. However, longitudinal studies with multiple time points can provide some insight into this question. Lersy et al. show that 79% of patients had partial or complete regression of abnormal brain MRI findings at 189 days follow-up (31). Furthermore, Chammas et al. showed a marked decreased in collicular hyperintensity at 3-month follow-up (18). It is more likely that this type of dynamic neuro-evolution would be due an acute insult rather than pre-existing chronic conditions. Likewise, Agarwal et al. demonstrated an increase in ventricle size at a 22-day follow-up MRI that is likely due to an acute infectious process rather than chronic processes like alcoholism or neurodegenerative diseases which progress over a longer period of time (15). COVID-19 does contribute to acute brain injury, however, the extent to which the findings reported in brain MRI papers is due to COVID-19 vs. comorbidities is difficult to assess. Future studies should utilize pre- and post-COVID MRI scans as well as matched controls to better determine the extent to which COVID-19 causes brain injury. The UK Biobank study of 785 participants is a good example of such a study (41).

Analysis of the association between MRI abnormalities and clinical findings provides an insight into the mechanism of brain injury in COVID-19. Given that ACE2 receptors and associated SARS-CoV-2 virions are expressed on brain endothelial cells, direct injury mediated by SARS-CoV-2 is theoretically possible (42). However, a direct mechanism of injury is highly unlikely given that only 1.6% (3/184) of patients across 9 studies in our meta-analysis were found to have to have SARS-CoV-2 RNA in their CSF via RT-PCR. Indeed, indirect mechanisms of brain injury seem more plausible, one of which is mechanical ventilation – a known contributor to various neurological injuries including intracranial hemorrhages, ischemic stroke, and hypoxic ischemic encephalopathy (43). This mechanism is supported by multiple papers in our analysis which show an association between mechanical ventilation and the presence of CMBs, WM microhemorrhages, and encephalopathy on MRI (7, 26, 32). It should be noted, however, that patients who do not undergo mechanical ventilation can still present with acute MRI abnormalities suggesting that while mechanical ventilation may contribute to the development neurological abnormalities, it is not the only mechanism at play. The cytokine storm hypothesis is another hypothesis supported by multiple papers which show an association between abnormal MRI findings and elevated inflammatory markers (14, 18, 32). Moreover, a non-specific inflammatory response is more consistent with the heterogenous presentation that is seen on brain MRI. Thrombosis is another potential mechanism for brain injury supported by the association of abnormal MRI findings with elevated D-Dimer, though this association is relatively non-specific (7, 26). Lastly, cerebral vasculitis is another possible mechanism (25). Overall, the mechanism of brain injury in COVID-19 is likely due to multiple, indirect mechanisms, including microvascular infarction and post-infarction hemorrhage.

In addition to possible mechanism of injury, associations between clinical findings and abnormal MRI may provide a predictive model for identifying patients who are likely to present with abnormal MRI findings. Napolitano et al. for example, have determined a CSF inflammatory profile in patients with cerebral microbleeds, though the invasiveness of a lumbar puncture is a large drawback to CSF profiling (32). Future studies should evaluate the possibility of creating such predictive models but using easier to obtain data points.

It is unlikely that specific brain injuries in COVID-19 contribute to acute neurocognitive dysfunction. Many papers in our analysis show an association between lower cognitive functioning and various acute brain MRI abnormalities but no specific imaging pattern has yet emerged. Indeed, these associations are likely the result of confounding bias due to critical care illness. Other modalities, such as EEG and neurocognitive testing, can be used to corroborate MRI findings. A similar conclusion is drawn in terms of long-term cognitive dysfunction, AKA ‘brain fog’ in COVID-19 patients (44, 45). A 7-month follow up study showed no difference in cognitive functioning between patients with and without MRI abnormalities suggesting that ‘brain fog’ cannot routinely be determine by MRI (22). Additional long-term MRI studies are needed to determine (1) whether ‘brain fog’ is due to neurological injury and (2) whether that injury can be identified on MRI analysis.

There are several limitations with this meta-analysis study. First, we chose to analyze only MRI imaging findings to assess the neurological complications of COVID-19 because MRI can detect a broad range of anatomical abnormalities with high sensitivity. CT and other brain imaging modalities should also be explored. Most of the studies included in this analysis did not have propensity-matched control groups and/or pre-COVID-19 brain MRI scans for comparison. Therefore, some findings may be attributable to pre-existing conditions rather than caused or exacerbated by COVID-19. There is publication bias as the patients with more severe COVID-19 disease are more likely to be reported in the literature. Finally, unintentional reporting bias could be present given that virtually all papers in this meta-analysis were retrospective studies.

## Conclusion

Improved understanding of the imaging findings associated with neurological signs and symptoms amongst COVID-19 patients and survivors will help to identify common neurological injuries, inform the care of at-risk patients, and understand the mechanism of neurological injury and the progression of brain effects of COVID-19. In this meta-analysis of the neurological MRI findings in COVID-19 patients, we report the incidence of any MRI abnormality to be 55%. The dynamic nature of these abnormalities suggests that the observed brain injury is, at least in part, the result of a SARS-CoV-2 related (para)infectious process rather than chronic comorbidities. Although the presentation of COVID-19 brain injury on MRI is diverse, most injuries appear to be of vascular origin. Moreover, analysis of the association between MRI abnormalities and clinical findings suggests that there are likely many mechanisms by which brain injury occurs in COVID-19. The use of these clinical associations to form predictive models for identifying patients likely to present with MRI abnormalities should be explored by future studies. These studies should also investigate the neurological and neurocognitive manifestations associated with brain MRI abnormalities. Brain MRI studies with longer follow-up intervals are needed to provide detailed assessment of the neurological sequelae of COVID-19. Brain MRI studies analyzing patients with mild COVID-19 are also necessary.

## Data availability statement

The original contributions presented in the study are included in the article/Supplementary material, further inquiries can be directed to the corresponding authors.

## Author contributions

MB: Conceptualization, Data curation, Formal analysis, Investigation, Methodology, Validation, Visualization, Writing – original draft, Writing – review & editing. BM: Conceptualization, Data curation, Methodology, Validation, Writing – review & editing. WH: Data curation, Visualization, Writing – original draft. MM: Writing – review & editing. TD: Conceptualization, Investigation, Methodology, Supervision, Validation, Writing – review & editing.
