# Supplementary material for: Brain MRI findings in severe COVID-19 patients: a meta-analysis
Source: Front Neurol. 2023 Oct 12;14:1258352. doi: 10.3389/fneur.2023.1258352 (PMC10602808; doi:10.3389/fneur.2023.1258352)
Supplement: Supplementary file 1 [file Data_Sheet_1.docx]

**PUBMED Search Strategy:**

((COVID*[Title/Abstract]) OR (SARS-CoV-2[Title/Abstract]) OR (coronavirus[Title/Abstract]))

AND

((neuro MRI[Title/Abstract]) OR (brain MRI[Title/Abstract]) OR (neurological MRI*[Title/Abstract]) OR (brain magnetic resonance imaging[Title/Abstract] OR (neuroimaging[Title/Abstract]))

**PRISMA Checklist**

| **Section and Topic** | **Item #** | **Checklist item** | **Location where item is reported** |
| --- | --- | --- | --- |
| **TITLE** | | |  |
| Title | 1 | Identify the report as a systematic review. | Title |
| **ABSTRACT** | | |  |
| Abstract | 2 | See the PRISMA 2020 for Abstracts checklist. | Supplementary Material |
| **INTRODUCTION** | | |  |
| Rationale | 3 | Describe the rationale for the review in the context of existing knowledge. | Page 1-2 |
| Objectives | 4 | Provide an explicit statement of the objective(s) or question(s) the review addresses. | Page 1-2 |
| **METHODS** | | |  |
| Eligibility criteria | 5 | Specify the inclusion and exclusion criteria for the review and how studies were grouped for the syntheses. | Page 2 |
| Information sources | 6 | Specify all databases, registers, websites, organisations, reference lists and other sources searched or consulted to identify studies. Specify the date when each source was last searched or consulted. | Page 2 |
| Search strategy | 7 | Present the full search strategies for all databases, registers and websites, including any filters and limits used. | Page 2 |
| Selection process | 8 | Specify the methods used to decide whether a study met the inclusion criteria of the review, including how many reviewers screened each record and each report retrieved, whether they worked independently, and if applicable, details of automation tools used in the process. | Page 2-3 |
| Data collection process | 9 | Specify the methods used to collect data from reports, including how many reviewers collected data from each report, whether they worked independently, any processes for obtaining or confirming data from study investigators, and if applicable, details of automation tools used in the process. | Page 2-3 |
| Data items | 10a | List and define all outcomes for which data were sought. Specify whether all results that were compatible with each outcome domain in each study were sought (e.g. for all measures, time points, analyses), and if not, the methods used to decide which results to collect. | Page 2 |
|  | 10b | List and define all other variables for which data were sought (e.g. participant and intervention characteristics, funding sources). Describe any assumptions made about any missing or unclear information. | Page 2 |
| Study risk of bias assessment | 11 | Specify the methods used to assess risk of bias in the included studies, including details of the tool(s) used, how many reviewers assessed each study and whether they worked independently, and if applicable, details of automation tools used in the process. | Page 2 |
| Effect measures | 12 | Specify for each outcome the effect measure(s) (e.g. risk ratio, mean difference) used in the synthesis or presentation of results. | N/A |
| Synthesis methods | 13a | Describe the processes used to decide which studies were eligible for each synthesis (e.g. tabulating the study intervention characteristics and comparing against the planned groups for each synthesis (item #5)). | Page 2 |
|  | 13b | Describe any methods required to prepare the data for presentation or synthesis, such as handling of missing summary statistics, or data conversions. | N/A |
|  | 13c | Describe any methods used to tabulate or visually display results of individual studies and syntheses. | N/A |
|  | 13d | Describe any methods used to synthesize results and provide a rationale for the choice(s). If meta-analysis was performed, describe the model(s), method(s) to identify the presence and extent of statistical heterogeneity, and software package(s) used. | N/A |
|  | 13e | Describe any methods used to explore possible causes of heterogeneity among study results (e.g. subgroup analysis, meta-regression). | N/A |
|  | 13f | Describe any sensitivity analyses conducted to assess robustness of the synthesized results. | N/A |
| Reporting bias assessment | 14 | Describe any methods used to assess risk of bias due to missing results in a synthesis (arising from reporting biases). | Supplementary Material |
| Certainty assessment | 15 | Describe any methods used to assess certainty (or confidence) in the body of evidence for an outcome. | N/A |
| **RESULTS** | | |  |
| Study selection | 16a | Describe the results of the search and selection process, from the number of records identified in the search to the number of studies included in the review, ideally using a flow diagram. | Fig. 1 |
|  | 16b | Cite studies that might appear to meet the inclusion criteria, but which were excluded, and explain why they were excluded. | N/A |
| Study characteristics | 17 | Cite each included study and present its characteristics. | Pages 3-8 |
| Risk of bias in studies | 18 | Present assessments of risk of bias for each included study. | N/A |
| Results of individual studies | 19 | For all outcomes, present, for each study: (a) summary statistics for each group (where appropriate) and (b) an effect estimate and its precision (e.g. confidence/credible interval), ideally using structured tables or plots. | N/A |
| Results of syntheses | 20a | For each synthesis, briefly summarise the characteristics and risk of bias among contributing studies. | Pages 3-8 |
|  | 20b | Present results of all statistical syntheses conducted. If meta-analysis was done, present for each the summary estimate and its precision (e.g. confidence/credible interval) and measures of statistical heterogeneity. If comparing groups, describe the direction of the effect. | N/A |
|  | 20c | Present results of all investigations of possible causes of heterogeneity among study results. | N/A |
|  | 20d | Present results of all sensitivity analyses conducted to assess the robustness of the synthesized results. | N/A |
| Reporting biases | 21 | Present assessments of risk of bias due to missing results (arising from reporting biases) for each synthesis assessed. | Page 17 |
| Certainty of evidence | 22 | Present assessments of certainty (or confidence) in the body of evidence for each outcome assessed. | Page 17 |
| **DISCUSSION** | | |  |
| Discussion | 23a | Provide a general interpretation of the results in the context of other evidence. | Page 14-17 |
|  | 23b | Discuss any limitations of the evidence included in the review. | Page 16-17 |
|  | 23c | Discuss any limitations of the review processes used. | Page 16-17 |
|  | 23d | Discuss implications of the results for practice, policy, and future research. | Page 14-17 |
| **OTHER INFORMATION** | | |  |
| Registration and protocol | 24a | Provide registration information for the review, including register name and registration number, or state that the review was not registered. | N/A |
|  | 24b | Indicate where the review protocol can be accessed, or state that a protocol was not prepared. | Figure 1 and Supplementary Material |
|  | 24c | Describe and explain any amendments to information provided at registration or in the protocol. | N/A |
| Support | 25 | Describe sources of financial or non-financial support for the review, and the role of the funders or sponsors in the review. | Reported to journal |
| Competing interests | 26 | Declare any competing interests of review authors. | Reported to journal |
| Availability of data, code and other materials | 27 | Report which of the following are publicly available and where they can be found: template data collection forms; data extracted from included studies; data used for all analyses; analytic code; any other materials used in the review. | N/A |

**PRISMA Abstract Checklist**

| **Section and Topic** | **Item #** | **Checklist item** | **Reported (Yes/No)** |
| --- | --- | --- | --- |
| **TITLE** | | |  |
| Title | 1 | Identify the report as a systematic review (or metanalysis) | Yes |
| **BACKGROUND** | | |  |
| Objectives | 2 | Provide an explicit statement of the main objective(s) or question(s) the review addresses. | Yes |
| **METHODS** | | |  |
| Eligibility criteria | 3 | Specify the inclusion and exclusion criteria for the review. | Yes |
| Information sources | 4 | Specify the information sources (e.g. databases, registers) used to identify studies and the date when each was last searched. | No |
| Risk of bias | 5 | Specify the methods used to assess risk of bias in the included studies. | No |
| Synthesis of results | 6 | Specify the methods used to present and synthesise results. | No |
| **RESULTS** | | |  |
| Included studies | 7 | Give the total number of included studies and participants and summarise relevant characteristics of studies. | Yes |
| Synthesis of results | 8 | Present results for main outcomes, preferably indicating the number of included studies and participants for each. If meta-analysis was done, report the summary estimate and confidence/credible interval. If comparing groups, indicate the direction of the effect (i.e. which group is favoured). | Yes |
| **DISCUSSION** | | |  |
| Limitations of evidence | 9 | Provide a brief summary of the limitations of the evidence included in the review (e.g. study risk of bias, inconsistency and imprecision). | No |
| Interpretation | 10 | Provide a general interpretation of the results and important implications. | Yes |
| **OTHER** | | |  |
| Funding | 11 | Specify the primary source of funding for the review. | N/A |
| Registration | 12 | Provide the register name and registration number. | N/A |

**Risk of Bias Assessment**

| **Paper** | **Author** | **Selection** | **Comparability** | **Outcome** |
| --- | --- | --- | --- | --- |
| Neurological complications in critical patients with COVID-19 | M.J. Abenza-Abildúa [6] | ★★ | - | ★★★ |
| Cerebral Microbleeds and Leukoencephalopathy in Critically Ill Patients With COVID-19 | Shashank Agarwal [7] | ★★★ | - | ★★★ |
| Retrospective Observational Study of Brain MRI Findings in Patients with Acute SARS-CoV-2 Infection and Neurologic Manifestations | Lydia Chougar [8] | ★★★ | - | ★★★ |
| Unusual Microbleeds in Brain MRI of Covid-19 Patients | Aikaterini Fitsiori [9] | ★★ | - | ★★★ |
| Delirium and encephalopathy in severe COVID-19: a cohort analysis of ICU patients | Julie Helms [10] | ★★★ | - | ★★★ |
| Brain MRI Findings in Patients in the Intensive Care Unit with COVID-19 Infection | Sedat G. Kandemirli [11] | ★★★ | - | ★★★ |
| Nervous System Involvement in Coronavirus Disease 2019: | Stefanos Klironomos [12] | ★★ | - | ★★★ |
| Neurologic and neuroimaging findings in patients with COVID-19 | Stephane Kremer [13] | ★★★ | - | ★★★ |
| Brain MRI Findings in Severe COVID-19: A  Retrospective Observational Study | Stephane Kremer [14] | ★★★ | - | ★★★ |
| Increase in Ventricle Size and the Evolution | Shashank Agarwal [15] | ★★ | ★★ | ★★★ |
| Brain MRI in SARS‐CoV‐2  pneumonia patients with newly  developed neurological  manifestations suggestive of brain  involvement | Batil Alonazi [16] | ★★★ | - | ★★★ |
| Clinical and Radiological Profiles of COVID-19 Patients with Neurological Symptomatology: A Comparative Study | Maria de Fatima Viana Vasco Aragao [17] | ★★ | - | ★★★ |
| Collicular Hyperactivation in Patients with COVID-19: A New Finding on Brain MRI and PET/CT | A. Chammas [18] | ★★ | - | ★★★ |
| Susceptibility-weighted imaging reveals cerebral microvascular injury in severe COVID-19 | John Conklin [19] | ★★ | - | ★★★ |
| Coronavirus Disease (COVID-19)-Related Disseminated Leukoencephalopathy: A Retrospective Study of Findings on Brain MRI | Colbey W. Freeman [20] | ★★ | - | ★★★ |
| Yield of Head Imaging in Ambulatory and Hospitalized Patients With SARS-CoV-2: A Multi-Center Study of 8675 Patients | Melanie R. F. Greenway [21] | ★★★ | - | ★★★ |
| Brain MRI and neuropsychological findings at long-term follow-up after COVID-19 hospitalisation: an observational cohort study | Lovisa Hellgren [22] | ★★ | - | ★★★ |
| Association of Clinical, Biological, and Brain Magnetic Resonance Imaging Findings With Electroencephalographic Findings for Patients With COVID-19 | Virginie Lambrecq [23] | ★★ | - | ★★★ |
| Abnormal MRI findings of the orbital or visual pathways in patients with severe COVID-19: Observations from the French multicenter COVID-19 cohort | Augustin Lecler [24] | ★★ | - | ★★★ |
| Cerebral vasculitis of medium-sized vessels as a possible mechanism of brain damage in COVID-19 patients | Francois Lersy [25] | ★★★ | - | ★★★ |
| Critical illness‐associated cerebral microbleeds for patients with severe COVID‐19: etiologic hypotheses | Francois Lersy [26] | ★★★ | - | ★★★ |
| Central Nervous System Injury in Patients With | Edith Fabiola Mendez Elizondo [27] | ★★ | - | ★★ |
| Distinct pattern of microsusceptibility changes on brain magnetic resonance imaging (MRI) in critically ill patients | Majda M. Thurnher [28] | ★★ | - | ★★★ |
| Long COVID-19: Objectifying most self-reported neurological symptoms | Julia Bungenberg [29] | ★★★ | - | ★★★ |
| Cognitive, EEG, and MRI features of COVID‐19 survivors: a 10‐month study | Giordano Cecchetti [30] | ★★★ | - | ★★★ |
| Evolution of Neuroimaging Findings in Severe COVID-19 Patients with Initial Neurological Impairment: An Observational Study | François Lersy [31] | ★★★ | - | ★★★ |
| Cerebral Microbleeds Assessment and Quantification in COVID-19 Patients With Neurological Manifestations | Angela Napolitano [32] | ★★★★ | ★★ | ★★★ |
| Early postmortem brain MRI findings in COVID-19 non-survivors | Tim Coolen [33] | ★★ | - | ★★★ |
| Disorders of Consciousness Associated With COVID-19 | David Fischer [34] | ★★★ | ★★ | ★★★ |
| Neurologic manifestations associated with COVID-19: a multicentre registry | Elodie Meppiel [35] | ★★★ | - | ★★★ |
| Neuroimaging Findings of Hospitalized Covid-19 Patients: A Canadian Retrospective Observational Study | Vibeeshan Jegatheeswaran [36] | ★★★ | - | ★★★ |
| Brain Imaging of Patients with COVID-19: Findings at an Academic Institution during the Height of the Outbreak in New York City | E. Lin [37] | ★★★ | - | ★★★ |
